# A novel algorithm for the treatment strategy for advanced epithelial ovarian cancer: consecutive imaging, frailty assessment, and diagnostic laparoscopy

**DOI:** 10.1186/s12885-017-3476-1

**Published:** 2017-07-12

**Authors:** Kyung Jin Eoh, Jung Won Yoon, Jung-Yun Lee, Eun Ji Nam, Sunghoon Kim, Sang Wun Kim, Young Tae Kim

**Affiliations:** Department of Obstetrics and Gynecology, Institute of Women’s Medical Life Science, Yonsei University College of Medicine, 50-1, Yonsei-ro, Seodaemun-gu, Seoul, 03722 Korea

**Keywords:** Epithelial ovarian cancer, Laparoscopy, Cytoreduction surgical procedures, Debulking surgical procedures

## Abstract

**Background:**

This study aimed to evaluate the perioperative outcomes and prognostic impact of the consecutive steps of imaging, frailty assessment, and diagnostic laparoscopy (DLS) in patients with advanced epithelial ovarian cancer (EOC).

**Methods:**

Patients diagnosed with EOC during 2012–2015 were analyzed retrospectively. Surgical and survival outcomes were compared between three treatment groups: patients without high tumor dissemination (HTD) who underwent primary debulking surgery (PDS group); patients with HTD who underwent DLS (DLS group); and patients with HTD diagnosed by cytological confirmation of malignancy followed by neoadjuvant chemotherapy (NACT group).

**Results:**

Of 181 patients, 85, 38, and 58 underwent PDS, DLS, and NACT, respectively. Among the 38 consecutive patients who initially underwent DLS, 6 were considered suitable for PDS; the remaining 32 were eligible for NACT followed by interval debulking surgery. The median operative times of debulking surgery in the PDS, DLS, and NACT groups were 365 min (interquartile range [IQR]: 216.5–476.5 min), 266.2 min (IQR: 160.3–193.5 min), and 339.0 min (IQR: 205–425 min; *P* = 0.042), respectively, with respective median estimated blood loss volumes of 962.2 mL (IQR: 300–1037.5 mL), 267.1 mL (IQR: 150–450 mL), and 861.7 mL (IQR: 150–1200 mL; *P* = 0.023). The DLS group had significantly reduced transfusion requirements and intensive care unit admission rates (*P* = 0.006). The Kaplan–Meier survival analysis indicated significantly poor PFS in the NACT group. However, there was no significant difference in OS among the three groups.

**Conclusions:**

The consecutive steps of imaging, frailty assessment, and DLS might facilitate rapid assessments of peritoneal disease extent and resectability; this novel algorithm might also be used to individualize treatment.

## Background

Epithelial ovarian cancer (EOC) remains a major cause of gynecologic cancer-related mortality because more than two-thirds of patients present with advanced disease at diagnosis [1, 2]. Two theoretical considerations have led cytoreductive surgery to be the recommended treatment for patients with advanced EOC: the physiological benefit of tumor excision and improved tumor perfusion with increased growth fraction [3, 4]. Optimal cytoreductive surgery is strongly emphasized because the presence of residual tumor after primary surgery is among the most important prognostic factors in patients with advanced EOC [5]. However, optimal cytoreduction is difficult to achieve, especially in the presence of extensive disease on the diaphragm or liver parenchyma, along the base of the small bowel mesentery, or in the lesser omentum or porta hepatis [6]. Moreover, the therapeutic value of surgery is questionable when the entire tumor mass cannot be resected [7]. Therefore, neoadjuvant chemotherapy (NACT) followed by interval debulking surgery (IDS) has become a useful therapeutic option for cases that are not eligible for complete primary debulking surgery (PDS) [8].

In the past 10 years, the role of laparoscopy in determining the possibility of primary optimal cytoreduction in patients with advanced EOC has been scrutinized [9–11]. Notably, Fagotti et al. advocated the use of a laparoscopy-based scoring system to evaluate the resectability of ovarian tumors [9–13]. However, there are questions regarding the benefits of laparoscopy for patients with advanced EOC, specifically in the identification of patient subgroup that will be best served by this strategy and determination of the most appropriate treatment strategy. Nonetheless, the application of diagnostic laparoscopy surgery (DLS) to all cases of advanced EOC appears to be problematic with regard to patient frailty and cost. And preoperative imaging has been suggested to be potentially useful in the prevention of unnecessary DLS.

Accordingly, the present study aimed to evaluate the perioperative outcomes and prognostic impact of the consecutive steps of imaging, frailty assessment, and DLS in patients with advanced EOC. The ultimate intent of this research was to propose an algorithm that would identify optimal candidates for DLS.

## Methods

### Study design

A retrospective study was performed. Patients who were diagnosed with EOC from March 2012 to March 2015 were enrolled in this study. The protocol recieved Institutional Review Board approval of the Yonsei University College of Medicine (No. 4–2017-0068) and was performed in accordance with the ethnical standards described in the Declaration of Helsinki. Preoperative clinical and radiological evaluations included chest radiography, pelvic ultrasonography, computed tomography (CT), and a serum carbohydrate antigen (CA) 125 level assessment. A gynecologic oncology team at a single institute conducted all procedures, and a dedicated radiologist at the same institute reviewed all data from preoperative imaging.

**Fig. 1:**
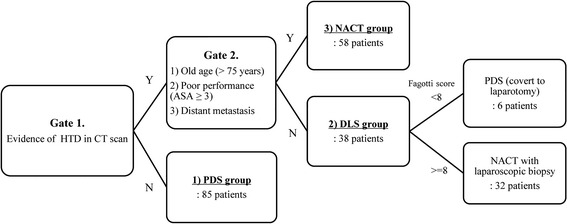
Flowchart of patient selection according to the proposed treatment algorithm for the consecutive use of imaging, frailty assessment, and DLS. HTD, high tumor dissemination; CT, computed tomography; ASA, American Society of Anesthesiologists physical status classification system; PDS, primary debulking surgery; NACT, neoadjuvant chemotherapy; DLS, diagnostic laparoscopy

A flow diagram of the patient selection process with a proposed treatment algorithm is presented in Fig. 1. High tumor dissemination (HTD) was defined as carcinomatosis, including bulky nodules (nodules >4 cm or plaques) on the diaphragm surface and mesentery or liver metastasis [14]. HTD was determined based on preoperative CT scanning and was assumed to be present when any of the following findings were suspected: (1) retroperitoneal lymph nodes >1 cm above the renal hilum; (2) diffuse small bowel adhesion/thickening; (3) perisplenic lesions >1 cm; (4) small bowel mesentery lesions >1 cm; (5) superior mesenteric artery lesion root >1 cm; (6) lesser sac lesion >1 cm; (7) diffuse peritoneal thickening >4 mm at the lateral colic gutters, anterior abdominal wall, diaphragm, and pelvic peritoneal reflections; (8) carcinomatosis with bulky nodules >4 cm; and (9) liver parenchymal metastasis.

**Fig. 2:**
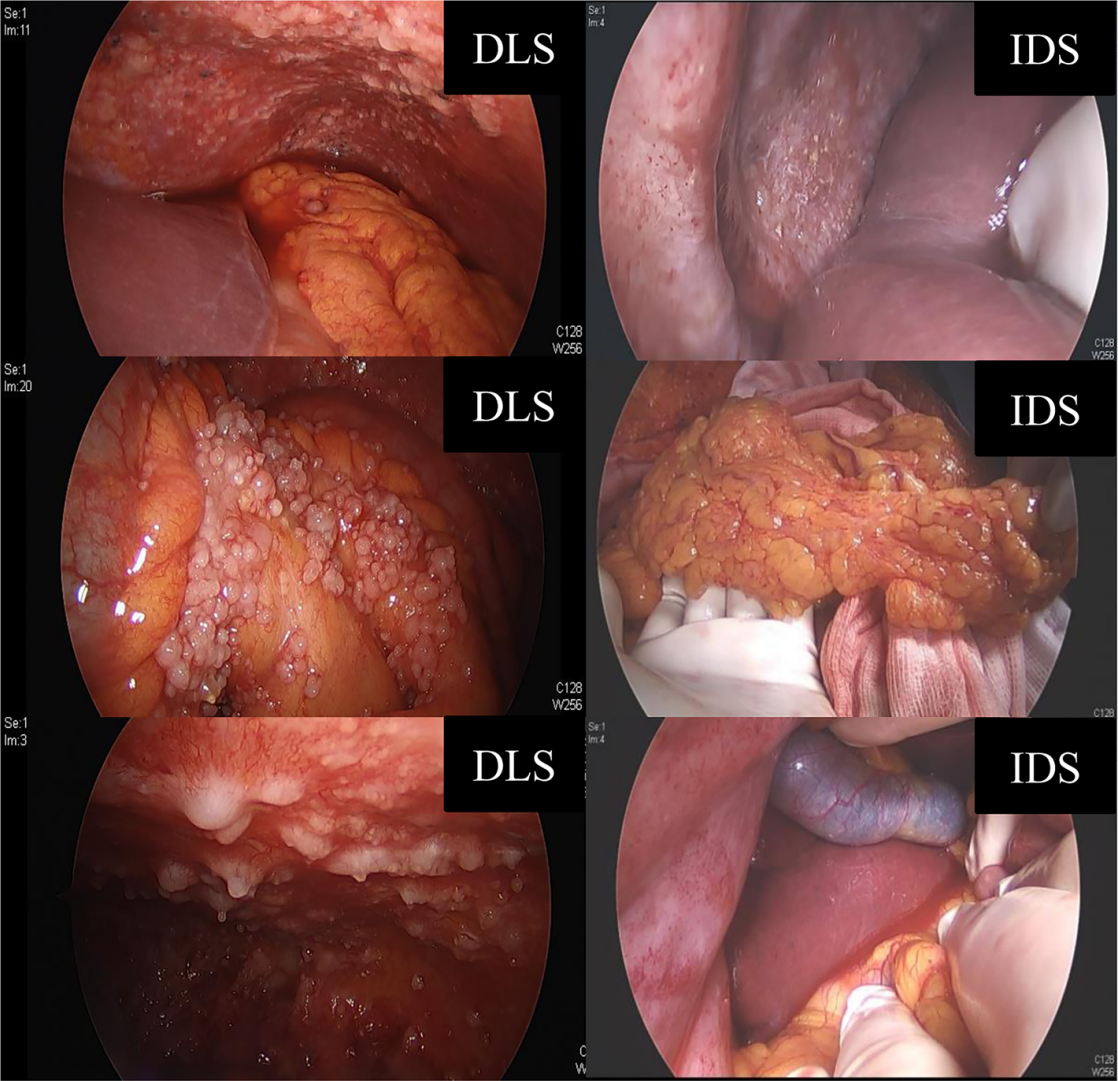
Surgical findings of DLS and IDS. A patient underwent DLS, which revealed a Fagotti score of 10. Neoadjuvant chemotherapy was administered 3 times, and the patient subsequently underwent IDS. DLS, diagnostic laparoscopy; IDS, interval debulking surgery

Patients were categorized into three groups according to the treatment intention. Those with no evidence of HTD were categorized into the PDS surgery group, and underwent primary staging laparotomy. Patients with evidence of HTD on CT scans were further divided into the following two groups according to a frailty assessment: (1) the neoadjuvant chemotherapy (NACT) group, in which patients underwent ascites cytology followed by NACT; and (2) the DLS group, in which patients underwent DLS to determine possibility of optimal debulking surgery. Patients with an older age (>75 years), with distant metastases, and with poor performance status (American Society of Anesthesiologists physical status classification system ≥3) were more likely to be categorized into the NACT group. In the DLS group, tumor resectability was evaluated using the Fagotti scoring system and consensus among surgeons who participated in the procedure. The following 7 parameters were assessed: (1) omental cake, (2) peritoneal carcinomatosis, (3) diaphragmatic carcinomatosis, (4) mesenteral retraction, (5) bowel infiltration, (6) stomach infiltration, and (7) superficial liver metastases [9]. Each parameter was assigned 2 points if present and 0 points otherwise. A conversion to laparotomy for PDS was considered when the laparoscopy-based score was less than 8 (Fig. 2).

Patients in the PDS, NACT, and DLS groups were compared with respect to their clinical features and surgical outcomes. Survival outcomes were also analyzed and compared among the 3 groups. Progression-free survival (PFS) was defined as the interval between the date that treatment was started and the date of documented disease progression or death from any cause. Overall survival (OS) was defined as the interval between the date treatment started and the date of death due to any cause. If a patient was lost to follow-up, that patient was censored at the last date of contact.

### Statistical analysis

IBM SPSS version 20 for Windows (SPSS Inc., Chicago, IL, USA) was used for the statistical analysis. The Kolmogorov–Smirnov test was used to verify standard normal distributional assumptions. Surgical outcomes were evaluated using an analysis of variance and the Kruskal–Wallis test. The chi-square test and Student’s t-test were used in the univariate analysis. Survival outcomes were determined through a Kaplan–Meier survival analysis. A *P*-value of <0.05 was considered statistically significant.

## Results

The characteristics of patients in this study are listed in Table 1. A total of 181 patients with FIGO stage IIIc and IV disease were included in the study. These patients were classified into the PDS (85 patients), DLS (38 patients), and NACT groups (58 patients). The patients in the NACT group were more likely to be older (*P* < 0.001), have poor performance (*P* < 0.001), and have distant metastasis (*P* < 0.001) compared with the other two groups.

**Table 1:** Patient characteristics

	PDS	DLS	NACT	P
	*n* = 85	*n* = 38	*n* = 58	
Age, mean (range)	55.8 (29–79)	50.8 (27–69)	59.7 (38–79)	<0.001
>75	5 (5.9%)	0 (0.0%)	5 (8.6%)	
ASA score				
1	16 (18.8%)	13 (34.2%)	6 (10.3%)	<0.001
2	44 (51.8%)	22 (57.9%)	26 (44.8%)	
3	24 (28.2%)	0 (0.0%)	26 (44.8%)	
BMI [IQR]	23.8 [20.9; 25.2]	24.1 [21.1; 25.4]	22.5 [20.6; 24.1]	0.236
CA125, U/mL (range)	1829.4 (44–22,743)	2191.7 (75–13,637)	3828.7 (102–17,094)	0.005
Stage				
IIIC	51 (60%)	31 (81.6%)	11 (19.0%)	<0.001
IV	34 (40%)	7 (18.4%)	47 (81.0%)	
Grade				
1	5 (5.9%)	2 (5.3%)	3 (5.2%)	0.708
2	28 (32.9%)	8 (21.1%)	11 (19.0%)	
3	45 (52.9%)	26 (68.4%)	39 (67.2%)	

Unless indicated otherwise, data are presented as n (%)

PDS, primary debulking surgery; DLS, diagnostic laparoscopic surgery; NACT, neoadjuvant chemotherapy; ASA, American Society of Anesthesiologists physical status classification system; BMI, body mass index; IQR, interquartile range; CA 125, carbohydrate antigen 125

A comparison of surgical outcomes is presented in Table 2. No DLS-related complications were reported. All the patients who received NACT underwent IDS after 2 to 4 cycles of NACT. The median debulking surgery operative times were 365.0 min (interquartile range [IQR]: 216.5–476.5 min) in the PDS group, 266.2 min (IQR: 160.3–193.5 min) in the DLS group, and 339.0 min (IQR: 205–425 min) in the NACT group (*P* = 0.042), with corresponding median estimated blood loss volumes of 962.2 mL (IQR: 300–1037.5 mL), 267.1 mL (IQR: 150–450 mL), and 861.7 mL (IQR: 150–1200 mL), respectively (*P* = 0.023). NACT group showed slightly shorter duration of surgery compared with PDS group, probably due to decreased tumor burden. The DLS group had significantly lower rates of transfusion necessity and intensive care unit admission (*P* = 0.016). The optimal debulking rates were not different among the three groups (PDS, 94.1%; DLS, 97.4%; NACT, 91.4%; *P* = 0.484).

**Table 2:** Surgical outcomes of cytoreductive surgery

	PDS	DLS	NACT	P
	*n* = 85	*n* = 38	*n* = 58	
OP time, min [IQR]	365.0 [216.5; 476.5]	266.2 [160.3; 193.5]	339.0 [205; 425]	0.042
Blood loss, mL [IQR]	962.2 [300; 1037.5]	267.1 [150; 450]	861.7 [150; 1200]	0.023
Transfusion	38 (44.7%)	9 (23.7%)	28(48.3%)	0.016
ΔHb [IQR]	1.0 [0; 1.98]	1.0 [0; 1.8]	0.1 [−0.5; 1.8]	<0.001
ICU admission	32 (37.6%)	4 (10.5%)	22 (37.9%)	0.006
Optimum	80 (94.1%)	37 (97.4%)	53 (91.4%)	0.484
Residual disease				
< 0.5 cm	70 (82.4%)	34 (89.5%)	50 (86.2%)	0.576
< 1 cm	10 (11.8%)	3 (7.9%)	3 (5.2%)	
< 2 cm	1 (1.2%)	1 (2.6%)	1 (1.7%)	
≥2 cm	4 (4.7%)	0 (0.0%)	4 (6.9%)	

Unless indicated otherwise, data are presented as n (%)

PDS, primary debulking surgery; DLS, diagnostic laparoscopic surgery; NACT, neoadjuvant chemotherapy; OP, operative; ΔHb, change in hemoglobin; IQR, interquartile range; ICU, intensive care unit

**Fig. 3:**
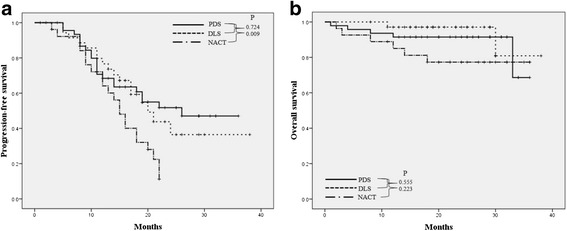
**a** Progression-free survival (PFS), and **b** overall survival (OS) of patients with advanced epithelial ovarian cancer (EOC), according to treatment intention

The median PFS and OS times for the PDS group were 16 months and 24 months, respectively, whereas the corresponding values for the DLS group were 16 months and 20 months, respectively. In addition, the median PFS and OS times for the NACT group werre 15 months and 24 months, respectively. The Kaplan–Meier survival analysis indicated significantly poor PFS in the NACT group (Fig. 3a). However, there was no significant difference in OS among the three groups (Fig. 3b).

An analysis of the Fagotti scores of patients initially intended to undergo DLS is presented in Table 3. All 38 patients had carcinomatosis, and omental cake and mesenteral retraction were observed in 28 (87.5%) and 27 patients (89.7%), respectively. Among these 38 consecutive patients, 6 were considered suitable for PDS, and the remaining 32 received NACT. The median Fagotti score of the 6 patients who converted to PDS was 4 (range: 4–10); all 6 patients underwent optimal debulking surgery. The remaining 32 patients who underwent NACT after DLS had a median Fagotti score of 8 (range: 4–14). Thirty-one out of 32 patients underwent optimal debulking surgery.

**Table 3:** Scoring of laparoscopic parameters among patients initially indicated for DLS

	PDS (*n* = 6)	No PDS (*n* = 32)
Fagotti score	4 (4–10)	8 (4–14)
(median, range)		
Omental cake	4 (66.7%)	28 (87.5%)
Peritoneal carcinomatosis	6 (100%)	32 (100%)
Diaphragm	2 (33.3%)	26(81.3%)
Mesenteral retraction	1 (16.7%)	27 (84.4%)
Bowel infiltration	0 (0%)	15 (46.9%)
Stomach infiltration	0 (0%)	7 (21.9%)
Liver surface	1 (16.7%)	3 (9.4%)
Optimal debulking rate of DS	6 (100.0%)	31 (96.9%)

Unless indicated otherwise, data are presented as n (%)

DLS, diagnostic laparoscopic surgery; PDS, primary debulking surgery; DS, debulking surgery

## Discussion

In the present study, we attempted to evaluate the usefulness of applying the consecutive steps of preoperative CT scans, patient frailty assessments, and DLS for determining the treatment strategy in advanced EOC. Preoperative CT scans appeared to be useful in the selection of proper candidates for primary PDS, thus avoiding unnecessary DLS in these patients. Among patients with evidence of HTD on imaging, patient frailty assessments were performed to select candidates for NACT in whom DLS could be omitted. In the remaining patients, DLS was found to facilitate the application of individualized treatment in which patients could avoid unnecessary laparotomies and surgical complications without sacrificing survival.

Surgeries performed by gynecologic oncologists play critical roles in the diagnosis, staging, and treatment of ovarian cancer [2]. To date, primary cytoreductive surgery followed by platinum-based adjuvant chemotherapy is a standard of disease management for patients who are medically stable and have no large effusions or parenchymal metastases. In addition, the feasibility of NACT followed by IDS for the treatment of FIGO stage IIIc or IV ovarian cancers was demonstrated in a prospective, randomized controlled trial [8]. In that study, the primary NACT group was not inferior to the primary surgery group with respect to median OS and PFS (OS, 29 vs. 30 months; PFS, 12 vs. 12 months). That study also emphasized the importance of complete resection of all macroscopic disease in both groups. Irrespective of whether the patient underwent primary surgery or primary NACT followed by IDS, the residual tumor size was inversely proportional to OS.

Therefore, the identification of patients with a high chance of achieving an optimal cytoreductive surgical outcome is an important step when determining treatment strategies for advanced EOC patients [14–16]. Unfortunately, several previously proposed models based on clinical or imaging techniques have failed to identify such patients preoperatively. Although previous studies have suggested the CA125 level (cut-off value, 500 International Units) as an indicator of the probability of an optimal cytoreduction, others have shown that these determinations have a low predictive value [17–19]. Imaging studies, including CT, magnetic resonance imaging, or positron emission tomography scans, have also been used to predict suboptimal resection [20–22]. However, a larger multi-institutional validation study reported accuracy rates as low as 34% for the ability of CT to predict suboptimal cytoreduction [23]. Positron emission tomography was also found to have limited positive predictive value [24].

Fagotti et al. proposed a scoring system based on laparoscopic findings to determine the possibility of optimal cytoreductive surgery. The items included in this system were the presence of an omental cake, peritoneal carcinomatosis, diaphragmatic carcinomatosis, mesenteric retraction, bowel infiltration, stomach infiltration, and liver metastases [11]. Each item was given 2 points if present, and a score > 8 predicted suboptimal surgery with a specificity of 100%, positive predictive value of 100%, and negative predictive value of 70%. This system was subsequently validated in an external cohort and also prospectively validated [12, 25].

However, decisions regarding the application of DLS to all patients with advanced ovarian cancer may be attributed to its poor cost-effectiveness and the complications associated with general anesthesia, especially in patients with an older age, poor performance status, or distant metastases. To avoid unnecessary DLS, we made decisions based on a combination of data from preoperative imaging and an assessment of the patient’s general condition. We noted that, although imaging studies have failed to predict the likelihood of suboptimal debulking surgery, these modalities could be effective to predict the likelihood of optimal cytoreduction [20, 21]. In the present study, we found that optimal surgeries could be performed in 94.1% of patients in the initially intended PDS group, whose optimal cytoreduction was highly predicted as possible in preoperative imaging study. Cytological analysis of ascites can provide pathologic confirmation and evidence to support a decision in favor of NACT, particularly for patients with a poor condition or distant metastases.

We proposed a treatment algorithm in Fig. 1. According to the algorithm, in patients found to have evidence of HTD (as described in the Methods) on preoperative imaging, decisions regarding DLS are based on the patient frailty assessment considering age, performance status, and the presence of distant metastases. NACT after a cytological ascites study, rather than DLS, was thought to be more appropriate for older patients with a poor performance status and/or distant metastases. On the other hand, the Fagotti scoring system can help to determine the likelihood of optimal cytoreduction in patients indicated for DLS. Specifically, if laparoscopic findings predict possible optimal cytoreduction, conversion to laparotomy for PDS is proposed. Otherwise, NACT after laparoscopic pathological confirmation is recommended, unless optimal debulking surgery appears possible.

## Conclusion

Herein, we attempted to address the benefits of laparoscopy for advanced EOC by determining the most appropriate treatment strategy and clarifying which patients would be best served by this strategy. Our data suggest that consecutive preoperative imaging, patient frailty assessment, and DLS may enable rapid assessment of the extent and resectability of peritoneal disease. It is also feasible to individualize treatment, thus avoiding unnecessary laparotomies and DLS and preventing surgical complications without decreasing survival. Additional prospective studies are necessary to validate the advantages of our proposed treatment algorithm, and continued attempts to individualize decisions concerning the most appropriate treatment strategy for advanced EOC are needed.

## Abbreviations

CA125: Carbohydrate antigen 125
CT: Computed tomography
DLS: Diagnostic laparoscopy
EOC: Epithelial ovarian cancer
HTD: High tumor dissemination
IQR: Interquartile range
NACT: Neoadjuvant chemotherapy
OS: Overall survival
PDS: Primary debulking surgery
PFS: Progression-free survival
